# Association of Nutritional Status at Diagnosis with Disease Course in Pediatric Inflammatory Bowel Disease

**DOI:** 10.3390/nu18193266

**Published:** 2026-10-03

**Authors:** Annalisa Morelli, Michela Caprarelli, Roberta Giusy Ruiz, Bianca Mazzoli, Alessia Salatto, Daniela Knafelz, Fiammetta Bracci, Sabrina Cardile, Giulia Bolasco, Chiara Maria Trovato, Paola De Angelis, Erminia Francesca Romeo, Antonella Diamanti

**Affiliations:** 1 Digestive Diseases and Nutritional Rehabilitation Unit, Bambino Gesù Children’s Hospital, Istituto di Ricovero e Cura a Carattere Scientifico (IRCCS), 00165 Rome, Italy; michela.caprarelli@gmail.com (M.C.); robertagiusy.ruiz@opbg.net (R.G.R.); biancamazzoli@gmail.com (B.M.); alessiasalatto1@gmail.com (A.S.); daniela.knafelz@opbg.net (D.K.); giulia.bolasco@opbg.net (G.B.);; 2 Department of Medicine, Surgery and Dentistry “Scuola Medica Salernitana”, Pediatrics Section, University of Salerno, 84081 Baronissi, Italy; 3 Department of Pediatrics, Università Cattolica del Sacro Cuore, Istituto di Ricovero e Cura a Carattere Scientifico (IRCCS), 00168 Rome, Italy; 4 Department of Pediatrics, University Federico II of Naples, Via Sergio Pansini 5, 80131 Naples, Italy; 5 Gastroenterology, Digestive Endoscopy and Nutrition Unit, Bambino Gesù Children’s Hospital, Istituto di Ricovero e Cura a Carattere Scientifico (IRCCS), 00165 Rome, Italy; paola.deangelis@opbg.net (P.D.A.); erminiafrancesca.romeo@opbg.net (E.F.R.)

**Keywords:** nutritional status, IBD, disease course

## Abstract

Background: Growth impairment is a major component of pediatric inflammatory bowel disease (IBD), yet the prognostic role of baseline nutritional status remains debated. We assessed the impact of nutritional status at diagnosis on subsequent medical and surgical disease course. Methods: We retrospectively studied 183 children with IBD (84 Crohn’s disease, 99 ulcerative colitis) followed for at least 18 months. Patients were classified at diagnosis using BMI Z-scores as underweight (n = 35), normal weight (n = 123), or overweight/obese (n = 25). Multivariable regression models—adjusted for diagnosis, biological sex, age at diagnosis, and extraintestinal manifestations/comorbidities—evaluated relapse burden, surgical resection, and longitudinal anthropometric recovery at last follow-up. Results: Baseline underweight status was not independently associated with a higher relapse burden or time to first relapse. A sensitivity analysis excluding surgically resected patients showed higher estimated relapse rates for underweight patients, though these did not reach statistical significance. In exploratory analyses, baseline underweight showed a borderline, non-significant association with subsequent surgical resection (adjusted OR 2.68, 95% CI 0.97–7.27; *p* = 0.056), warranting validation in larger prospective cohorts. Exploratory VEO-IBD analyses were limited by small subgroup size; within UC, a higher crude resection frequency was observed in VEO-UC than in older-onset UC (15.0% vs. 1.3%), but this finding should be interpreted cautiously. At follow-up, 85.7% of initially underweight patients reached normal weight. Higher baseline BMI Z-score was independently associated with higher follow-up BMI Z-score, whereas therapeutic escalation and total relapse burden were not independently associated with follow-up BMI Z-score. Persistent stunting was very rare (1.9%), precluding multivariable analysis. Conclusions: Baseline underweight showed a suggestive but non-significant association with subsequent surgical resection, warranting confirmation in larger prospective cohorts, while it was not independently associated with medical disease instability. Substantial weight recovery occurred during pediatric IBD follow-up. Baseline BMI Z-score was associated with BMI Z-score at follow-up, although substantial between-patient variability remained unexplained.

## 1. Introduction

Pediatric inflammatory bowel diseases (PIBD), including Crohn’s disease (CD) and ulcerative colitis (UC), are chronic, relapsing, immune-mediated disorders with onset during childhood or adolescence. Compared with adult-onset IBD, pediatric disease often presents with extensive intestinal involvement and has distinctive consequences for growth, pubertal development, bone health, and nutritional status [1]. These aspects are particularly relevant because childhood and adolescence are critical periods of somatic development, during which chronic inflammation, reduced dietary intake, malabsorption, and increased metabolic requirements may interfere with both weight gain and linear growth [2].

Nutritional impairment is therefore a major component of the pediatric IBD phenotype. Weight and growth deficits have traditionally been reported more frequently in CD than in UC, although the nutritional spectrum of PIBD is increasingly heterogeneous and includes not only undernutrition but also overweight and obesity [2,3,4,5]. Anthropometric status at diagnosis may reflect the cumulative nutritional and inflammatory burden preceding treatment and may also differ according to disease phenotype, age at onset, and other clinical characteristics. However, whether these baseline differences provide clinically meaningful information on the subsequent course of disease remains uncertain.

Several studies have suggested a potential prognostic role of nutritional status at diagnosis. In particular, both low and high BMI have been associated with a more severe pediatric IBD course, including increased disease activity, exacerbations, hospitalization, and treatment escalation, leading to the hypothesis of a U-shaped relationship between BMI and adverse outcomes [6,7]. Accordingly, although BMI Z-score was retained as a continuous exposure in the primary prognostic models, possible non-linearity was also evaluated in secondary analyses using clinically defined nutritional categories and a quadratic BMI Z-score specification. Nevertheless, findings across pediatric cohorts have not been uniform, particularly regarding overweight and obesity [8]. Moreover, medical disease instability and the need for surgical resection represent distinct clinical outcomes and may not share the same relationship with baseline anthropometric status. Growth impairment and other markers of severe disease have also been identified as potential indicators of a complicated clinical course, particularly in pediatric CD [9,10,11].

Nutritional status is also dynamic over time. Control of intestinal inflammation and appropriate nutritional management may allow substantial recovery in weight and BMI, whereas persistent disease activity, treatment exposure, or other biological factors may limit catch-up. Linear growth may follow a different trajectory from weight-related parameters because it is additionally influenced by endocrine pathways, disease duration, corticosteroid exposure, and pubertal development [2,12,13,14]. The extent to which relapse burden and therapeutic escalation independently contribute to long-term nutritional recovery remains incompletely defined. Very early-onset IBD (VEO-IBD), conventionally defined as disease diagnosed before 6 years of age, represents an additional source of clinical and biological heterogeneity and may represent a distinct pathophysiological entity (including monogenic defects or immune dysregulation in selected patients) characterized by severe phenotypes, which might independently drive profound early growth failure and treatment refractoriness, thus potentially altering the baseline anthropometric-clinical relationship observed in older pediatric cohorts [15].

The present study therefore aimed to evaluate whether baseline BMI Z-score and anthropometric categories were associated with subsequent relapse burden, time to first relapse, therapeutic escalation, or surgical resection. In addition, we established several secondary and exploratory objectives: (1) to characterize anthropometric status at diagnosis and identify associated clinical factors; (2) to examine clinical outcomes according to age at diagnosis, with particular attention to the VEO-IBD phenotype, which may represent biologically distinct and more severe disease driving early growth failure; (3) to describe longitudinal changes in BMI, weight, linear growth, and stunting during follow-up; and (4) to identify clinical and treatment-related factors associated with anthropometric recovery at the last follow-up assessment.

## 2. Materials and Methods

### 2.1. Study Design and Cohort Selection

A retrospective cohort study was conducted including patients with inflammatory bowel disease (IBD) followed at our Center. Patients who underwent a follow-up assessment between May 2025 and April 2026 were screened for eligibility. Inclusion criteria were:

Pediatric age at the time of diagnosis (defined as ≤18 years of age);

Diagnosis formalized at our Center;

A minimum follow-up duration of 18 months. This temporal threshold was chosen a priori to ensure sufficient observation time to reliably capture subsequent relapse burden, therapeutic escalation, and meaningful longitudinal anthropometric changes.

Based on these criteria, 208 patients were excluded from the analysis: 92 patients had an insufficient follow-up duration (<18 months, having been diagnosed from January 2025 onwards), and 116 patients were excluded because their initial diagnosis was established elsewhere prior to referral, resulting in unavailable baseline auxological parameters. The final analytical cohort comprised the remaining 183 patients.

For each patient, data were collected from the time of diagnosis and baseline assessment (T0) to the last available follow-up visit at our Center (T1). The study end date corresponded to the last available visit before 30 April 2026. The final analytical cohort comprised 183 patients.

Baseline clinical data retrieved from medical records included diagnosis, biological sex, age at diagnosis, and the presence of EIMs or comorbidities. The composite variable ‘EIMs/comorbidities’ included the presence of either extraintestinal manifestations directly related to IBD or other concurrent chronic conditions present at diagnosis. Extraintestinal manifestations specifically considered were peripheral arthritis, erythema nodosum, aphthous stomatitis, uveitis, primary sclerosing cholangitis, osteopenia/osteoporosis, iron-deficiency anemia and neuropsychiatric alterations. Comorbidities primarily encompassed other immune-mediated or systemic disorders, such as celiac disease, autoimmune thyroiditis, psoriasis, vitiligo and asthma.

Auxological parameters were systematically recorded by qualified personnel at T0 and T1 using calibrated digital scales and wall-mounted stadiometers. Nutritional status was standardized by calculating standard deviation scores (Z-scores) for weight, height, and body mass index (BMI) based on the Centers for Disease Control and Prevention (CDC) growth charts, using the Peditools software [16].

At diagnosis, nutritional status was classified according to BMI-for-age Z-score as underweight (Z-score < −2), normal weight (Z-score ≥ −2 to ≤+1), overweight (Z-score > +1 to ≤+2), or obese (Z-score > +2). For analyses in which the upper categories contained few observations, overweight and obesity were combined into a single category.

Stunting was defined as a height-for-age Z-score < −2. Stratification by age at diagnosis identified the subpopulation affected by very early-onset IBD (VEO-IBD), conventionally defined by an age < 6 years at diagnosis.

The study was conducted in accordance with the Declaration of Helsinki, and the protocol was approved by the Clinical Research Ethics Committee of Città della Salute e della Scienza, Turin, Italy (14 February 2020; protocol number 0016409).

### 2.2. Clinical and Nutritional Outcomes

Clinical disease course during follow-up was characterized by relapse burden, time to first relapse, therapeutic escalation, and surgical resection. A clinical relapse was defined by evidence of active disease according to clinical, biochemical, or endoscopic criteria. Specifically, a relapse was adjudicated in the presence of a Pediatric Crohn’s Disease Activity Index (PCDAI) >12.5, a Pediatric Ulcerative Colitis Activity Index (PUCAI) >10, or evidence of active disease at endoscopy. Importantly, an isolated elevation in fecal calprotectin (>250 µg/g) was not considered sufficient to define a relapse on its own; it strictly required corroborating clinical symptoms or endoscopic evidence of active inflammation. Furthermore, a new relapse was distinguished from persistent disease activity by the requirement of a previously documented period of clinical remission prior to the new event. Relapse burden was quantified as the total number of relapses recorded during follow-up; relapse rate was additionally expressed as the number of relapses per person-year of follow-up. Time to first relapse was calculated from diagnosis to the first recorded relapse.

Therapeutic escalation was defined according to the treatment-escalation categories recorded in the clinical database and reflected the introduction of additional maintenance therapy following inadequate response or failure to sustain remission. Specifically, the database categorized escalation into three main approaches: (1) introduction of immunomodulators (e.g., azathioprine or methotrexate); (2) initiation of first-line biologic therapy; and (3) switch to a second-line biologic or small molecule. For the purpose of the present study, to robustly capture the overall need for medical step-up as a marker of disease instability and to preserve statistical power in the multivariable models, these categories were collapsed into a single composite binary outcome. Consequently, therapeutic escalation in our analyses is not synonymous exclusively with biologic therapy. Surgical resection was defined as partial or total intestinal resection performed during follow-up.

Longitudinal anthropometric outcomes were assessed by comparing BMI and weight Z-scores between diagnosis (T0) and the last available follow-up visit (T1). Changes in these parameters were calculated as T1 minus T0. Nutritional status at T1 was also classified categorically, allowing assessment of transitions between underweight, normal weight, and overweight/obese status during follow-up. For patients who had reached adulthood by the T1 assessment, age-specific pediatric Z-scores were no longer applicable; therefore, standard adult BMI cut-offs were used to define their nutritional categories at follow-up (underweight: BMI < 18.5 kg/m^2^; normal weight: BMI 18.5–24.9 kg/m^2^; overweight/obese: BMI ≥ 25.0 kg/m^2^).

Linear growth was evaluated using height-for-age Z-scores at T0 and T1. Stunting was defined as a height-for-age Z-score < −2. Persistent stunting was defined as stunting at both T0 and T1, while recovery from stunting and new-onset stunting were defined according to the corresponding change in height-status category between the two assessments.

### 2.3. Statistical Analysis

Continuous variables were summarized as median and interquartile range (IQR), while categorical variables were reported as absolute frequencies and percentages. Comparisons between independent groups were performed using the Wilcoxon rank-sum test for continuous variables and Fisher’s exact test for categorical variables. When more than two independent groups were compared, non-parametric rank-based procedures were used as appropriate. Longitudinal analyses of anthropometric variables were restricted to patients with the corresponding measurement available at both T0 and T1. Regression models were fitted using complete cases for all variables included in the respective model.

To investigate factors associated with nutritional status at diagnosis, baseline BMI Z-score was analyzed as a continuous outcome using multivariable linear regression. In the overall cohort, the model included diagnosis (Crohn’s disease [CD] vs. ulcerative colitis [UC]), biological sex, age at diagnosis, and the presence of extraintestinal manifestations (EIMs) or comorbidities. Because anatomical disease extent is defined differently in CD and UC, its association with baseline BMI Z-score was not evaluated in a single pooled model. Instead, additional disease-specific multivariable linear regression models were fitted separately for CD and UC, including the corresponding disease-extent categories together with biological sex, age at diagnosis, and EIMs/comorbidities. Results were expressed as regression coefficients (β) with 95% confidence intervals (CI).

The prognostic value of baseline nutritional status for subsequent medical disease course was evaluated using separate models for relapse burden, time to first relapse, and therapeutic escalation. The total number of relapses recorded during follow-up was analyzed using negative binomial regression. To account for differences in observation time between patients, the logarithm of follow-up duration was included as an offset, and results were reported as incidence rate ratios (IRR). Time to first relapse was analyzed using Cox proportional hazards regression, with effect estimates expressed as hazard ratios (HR). The proportional-hazards assumption was formally assessed for the fitted Cox model. Therapeutic escalation was analyzed using multivariable logistic regression and expressed as odds ratios (OR). In these models, baseline BMI Z-score was the main exposure, with adjustment for diagnosis, biological sex, age at diagnosis, and EIMs/comorbidities; follow-up duration was additionally included as a covariate in the therapeutic-escalation model.

To assess whether the prognostic relationship depended on clinically defined nutritional categories rather than on BMI Z-score as a continuous measure, secondary analyses replaced baseline BMI Z-score with nutritional status classified as normal weight, underweight, or overweight/obese. Relapse-free survival according to baseline nutritional category was additionally described using Kaplan–Meier curves and compared using the log-rank test. Because previous studies have suggested a possible U-shaped association between BMI and adverse IBD outcomes, potential non-linearity was additionally assessed by refitting the relapse-rate, time-to-first-relapse, and therapeutic-escalation models with both linear and quadratic terms for baseline BMI Z-score. The quadratic models were compared with the corresponding linear models using likelihood-ratio tests. As a sensitivity analysis, these comparisons were repeated after exclusion of the single observation with an extreme baseline BMI Z-score (absolute Z-score > 6). These analyses were considered secondary. In the primary relapse analyses, patients who underwent surgical resection remained in the analytical cohort. For the relapse-rate analysis, all recorded relapses from diagnosis to the last available follow-up visit (T1) were included, and the entire observation period from diagnosis to T1 contributed person-time, irrespective of subsequent surgery. For the time-to-first-relapse analysis, patients were followed from diagnosis to the first recorded relapse or, if no relapse occurred, were censored at T1; surgical resection was not treated as a censoring event in the primary analysis. Because surgery may alter subsequent relapse ascertainment, both analyses were repeated as sensitivity analyses after excluding patients who underwent surgical resection during follow-up. Exact dates of surgical resection were not available in the analytical dataset, precluding a reliable sensitivity analysis censoring follow-up at the date of surgery. This sensitivity analysis therefore included only patients who did not undergo surgical resection during follow-up.

The association between baseline nutritional status and subsequent surgical resection was assessed separately from medical disease-course outcomes. Crude differences in surgical proportions across nutritional categories were evaluated using Fisher’s exact test. Because the number of surgical events was limited and some nutritional categories contained few events, adjusted analyses were performed using Firth bias-reduced logistic regression. In the overall cohort, the model included baseline nutritional status, diagnosis, age at diagnosis, biological sex, and follow-up duration. A separate CD-specific model was fitted including nutritional status, age at diagnosis, biological sex, and follow-up duration. Normal weight was used as the reference category. Secondary analyses examined BMI Z-score categories directly (<−2, −2 to +2, and >+2), baseline height Z-score as a continuous anthropometric predictor, and, within the CD subgroup, the additional contribution of anatomical disease extent. Adjusted associations were expressed as ORs with 95% CIs. Given the limited number of patients with VEO-IBD, all analyses according to VEO-IBD status were considered exploratory and were interpreted cautiously. Disease-specific analyses were additionally conducted separately within CD and UC, with particular caution for VEO-CD because only five patients were included in this subgroup.

Age-at-diagnosis analyses focused on the VEO-IBD phenotype, defined as diagnosis before 6 years of age. Patients with VEO-IBD were compared with those diagnosed at age ≥ 6 years using the Wilcoxon rank-sum test for continuous variables and Fisher’s exact test for categorical variables. Relapse rates were compared using negative binomial regression with the logarithm of follow-up duration as an offset. Disease-specific analyses were also conducted separately within CD and UC and were considered exploratory, particularly in CD because of the small number of patients with VEO-CD. In the overall cohort, adjusted associations between VEO-IBD status and subsequent outcomes were examined using negative binomial regression for relapse rate, logistic regression for therapeutic escalation, and Firth bias-reduced logistic regression for surgical resection. The relapse-rate model was adjusted for diagnosis and biological sex, with follow-up duration included as an offset, whereas the therapeutic-escalation and surgical-resection models were adjusted for diagnosis, biological sex, and follow-up duration.

Longitudinal changes in growth parameters were assessed by comparing paired measurements obtained at T0 and T1. Changes in BMI, weight, and height Z-scores were calculated as T1 minus T0 and analyzed using the paired Wilcoxon signed-rank test. Nutritional-category transitions between diagnosis and follow-up were summarized using transition counts and row percentages. The change in underweight prevalence between T0 and T1 was evaluated using an exact paired test based on discordant observations. Height-status transitions were analyzed using the same framework. Stunting was defined as a height-for-age Z-score < −2, and persistent stunting was defined as stunting at both T0 and T1. Because only three patients met the definition of persistent stunting, no multivariable model of factors associated with persistent stunting was fitted.

Factors associated with anthropometric status at the last follow-up were investigated using a multivariable linear regression model with BMI Z-score at T1 as the dependent variable and baseline BMI Z-score included as a covariate. This ANCOVA-type approach was used to evaluate follow-up nutritional status while accounting for differences already present at diagnosis. The model additionally included total relapse burden, therapeutic escalation, surgical resection during follow-up, diagnosis, biological sex, age at diagnosis, EIMs/comorbidities, and follow-up duration. Ordinary least-squares β coefficients were retained as point estimates, while 95% CIs and *p*-values were calculated using HC3 heteroskedasticity-robust standard errors.

Several sensitivity and exploratory analyses were conducted for this follow-up growth model. The model was repeated after exclusion of the single observation with an extreme baseline BMI Z-score (absolute Z-score > 6) and after restriction to patients aged <18 years at T1. Relapse burden was alternatively expressed as the number of relapses per person-year rather than as the total number of relapses. Potential non-linearity in the relationship between relapse burden and BMI Z-score at T1 was explored by adding a quadratic relapse term and, separately, by categorizing relapse burden as 0, 1, 2–5, or >5 relapses. These analyses were considered secondary and were used to assess the robustness and functional form of the primary associations.

All statistical tests were two-sided, and *p*-values < 0.05 were considered statistically significant. Effect estimates are reported with 95% CIs. No separate category of “statistical trend” was defined for *p*-values between 0.05 and 0.10. Statistical analyses and graphical visualizations were performed using the R software environment for statistical computing and graphics (version 4.6.1, R Foundation for Statistical Computing, Vienna, Austria).

## 3. Results

A total of 183 patients (102 males, 56%; 81 females, 44%) were included, comprising 84 patients with Crohn’s disease (CD) and 99 patients with ulcerative colitis (UC). The median age at diagnosis was 12.0 years (IQR 9.0–14.0 years), and the median follow-up duration was 4.27 years (IQR 2.65–5.79 years). Demographic and clinical characteristics of the cohort are summarized in Table 1. Patients with CD had lower BMI and weight Z-scores at diagnosis than patients with UC, whereas height Z-scores were comparable between disease groups.

### 3.1. Factors Associated with Anthropometric Status at Diagnosis

Multivariable linear regression analysis showed that diagnosis and age at diagnosis were independently associated with baseline BMI Z-score. In the overall cohort, patients with UC had a significantly higher BMI Z-score than patients with CD after adjustment for age, biological sex, and the presence of extraintestinal manifestations (EIMs) or comorbidities (β = 0.66, 95% CI 0.20 to 1.12, *p* = 0.005). Older age at diagnosis was also associated with a modestly higher BMI Z-score (β = 0.06 per year, 95% CI 0.01 to 0.12, *p* = 0.028). In contrast, biological sex (β = −0.31, 95% CI −0.77 to 0.15, *p* = 0.181) and the presence of EIMs or comorbidities (β = −0.01, 95% CI −0.47 to 0.44, *p* = 0.951) were not significantly associated with baseline BMI Z-score (Table 2).

Because disease extent is defined differently in CD and UC, its association with baseline nutritional status was subsequently evaluated separately within each disease phenotype. Among patients with CD, disease extent was not significantly associated with baseline BMI Z-score (global *p* = 0.603). Compared with colonic/pancolitis disease, neither ileal involvement (β = 0.01, 95% CI −0.88 to 0.91, *p* = 0.975) nor upper gastrointestinal involvement (β = 0.35, 95% CI −0.62 to 1.32, *p* = 0.475) was associated with baseline BMI Z-score. Biological sex, age at diagnosis, and EIMs/comorbidities were also not significantly associated with nutritional status within the CD subgroup.

Similarly, among patients with UC, extensive/colonic disease was not significantly associated with baseline BMI Z-score compared with distal disease (β = −0.49, 95% CI −1.28 to 0.31, *p* = 0.230). Older age at diagnosis, however, remained significantly associated with a higher BMI Z-score (β = 0.09 per year, 95% CI 0.01 to 0.17, *p* = 0.021). No significant associations were observed for biological sex (β = −0.36, *p* = 0.286) or EIMs/comorbidities (β = 0.37, *p* = 0.283).

**Figure 1:**
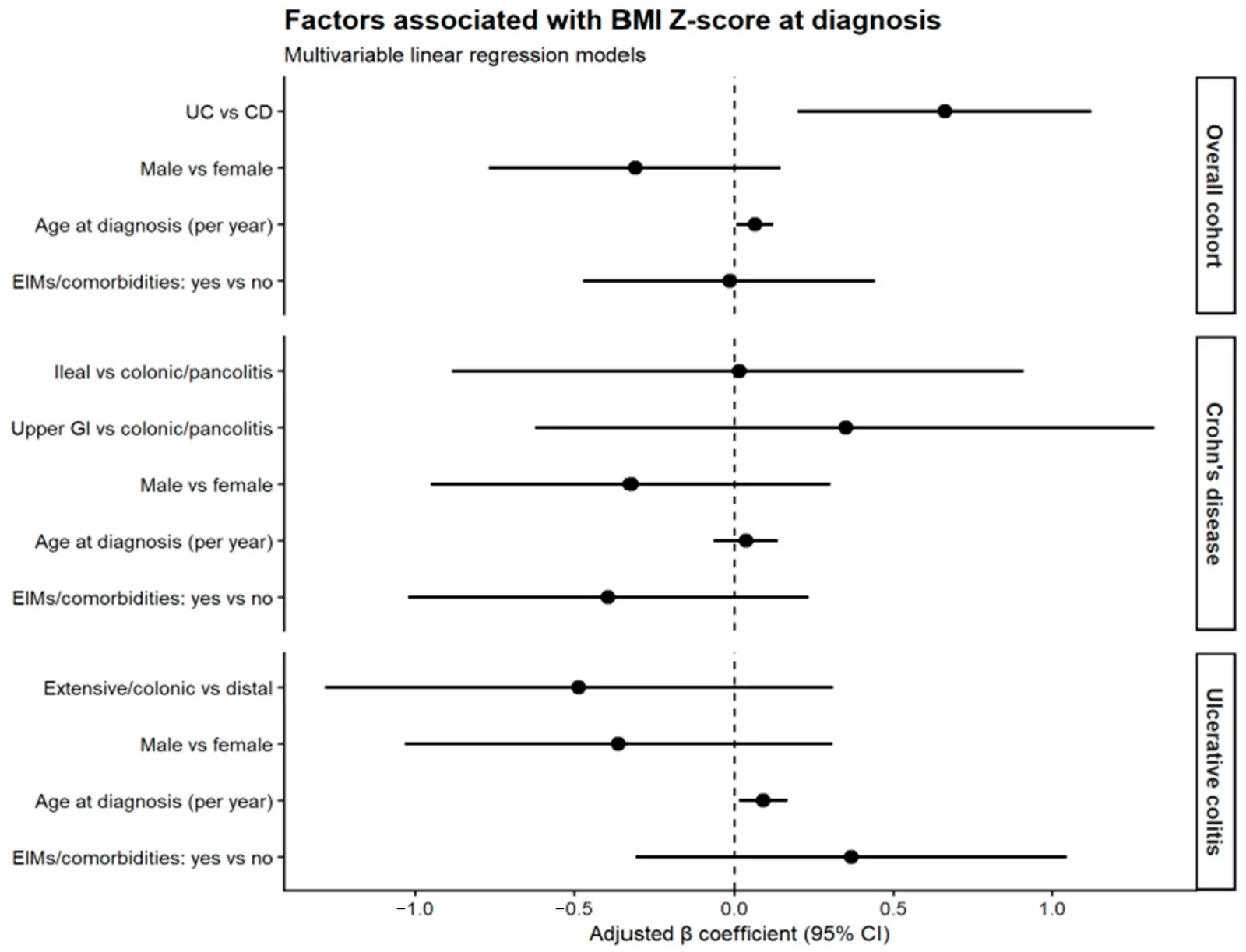
Adjusted associations between clinical characteristics and BMI Z-score at diagnosis. Forest plot showing adjusted β coefficients and 95% confidence intervals from multivariable linear regression models for the overall cohort and separately for patients with Crohn’s disease and ulcerative colitis. The dashed vertical line at β = 0 represents the null association; positive coefficients indicate higher baseline BMI Z-scores, whereas negative coefficients indicate lower BMI Z-scores.

The adjusted estimates and corresponding 95% confidence intervals across the three models are shown in Figure 1.

### 3.2. Prognostic Value of Baseline Nutritional Status for the Medical Disease Course

Baseline BMI Z-score was not significantly associated with subsequent medical disease course. In negative binomial regression accounting for follow-up duration, each 1-unit increase in baseline BMI Z-score was not associated with relapse rate (IRR = 0.94, 95% CI 0.84 to 1.05, *p* = 0.221). Similarly, baseline BMI Z-score was not associated with time to first relapse in the Cox proportional hazards model (HR = 0.93, 95% CI 0.83 to 1.05, *p* = 0.234), nor with the likelihood of therapeutic escalation (OR = 1.11, 95% CI 0.90 to 1.38, *p* = 0.351). The main prognostic estimates are summarized in Table 3.

In contrast, disease phenotype was associated with subsequent relapse burden. After adjustment for baseline BMI Z-score, biological sex, age at diagnosis, and EIMs/comorbidities, patients with UC had a higher relapse rate than patients with CD (IRR = 2.32, 95% CI 1.65 to 3.27, *p* < 0.001). Older age at diagnosis was also associated with a modestly higher relapse rate (IRR = 1.07 per year, 95% CI 1.03 to 1.11, *p* < 0.001), whereas biological sex and EIMs/comorbidities were not significantly associated with relapse rate.

Consistently, UC was associated with a higher hazard of first relapse than CD in the Cox model (HR = 1.69, 95% CI 1.16 to 2.48, *p* = 0.007). No significant associations with time to first relapse were observed for biological sex, age at diagnosis, or EIMs/comorbidities. The proportional hazards assumption was not violated (global *p* = 0.132).

**Figure 2:**
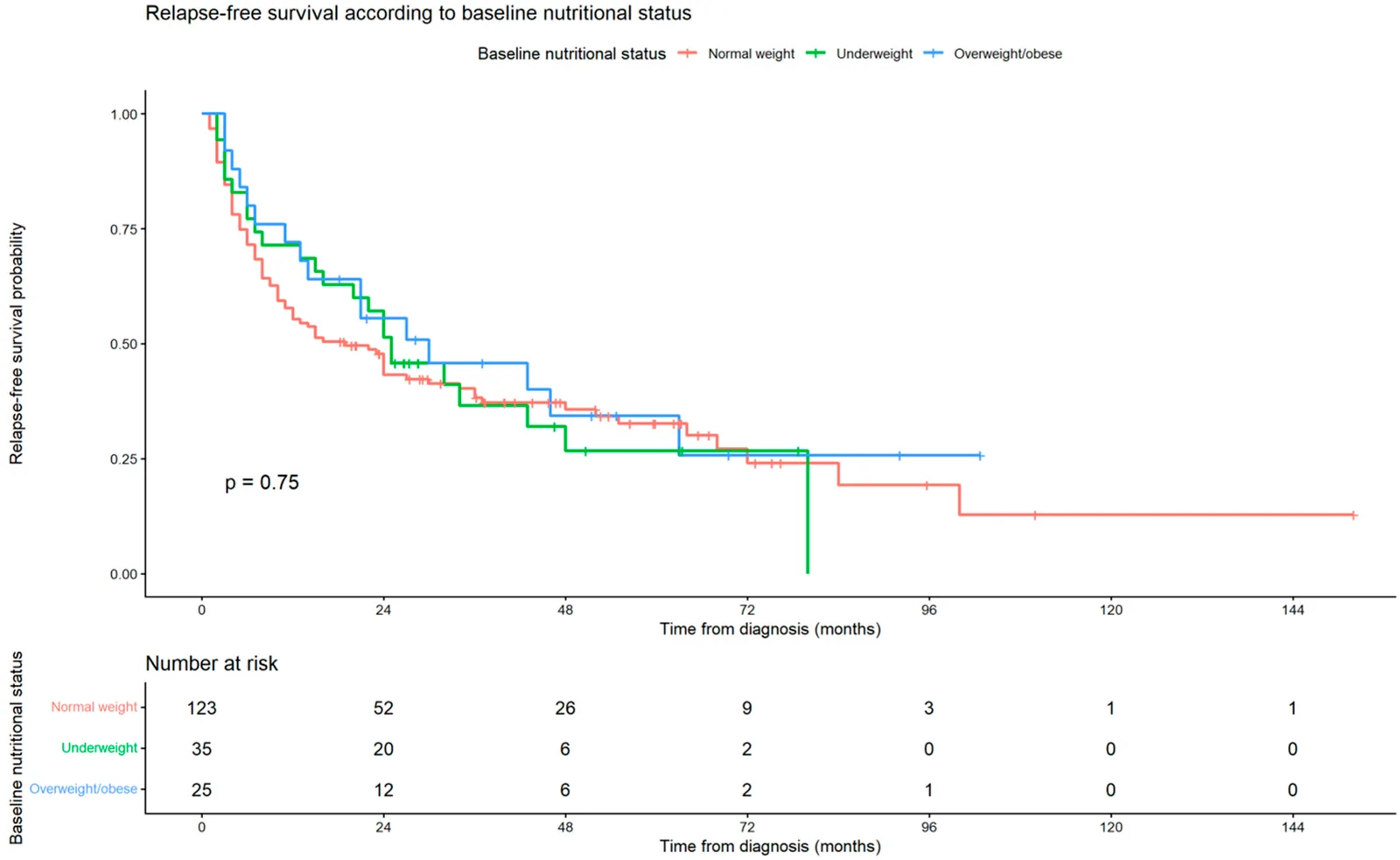
Relapse-free survival according to baseline nutritional status. Kaplan–Meier curves showing relapse-free survival from diagnosis according to baseline nutritional category (normal weight, underweight, and overweight/obese). Differences between groups were assessed using the log-rank test (*p* = 0.75). Tick marks indicate censored observations, and the table below the curves reports the number of patients at risk over time.

Secondary analyses treating baseline nutritional status categorically yielded consistent findings. Compared with patients of normal weight, neither underweight nor overweight/obesity was significantly associated with relapse rate, time to first relapse, or therapeutic escalation. For time to first relapse, the adjusted HR was 1.02 (95% CI 0.64 to 1.64, *p* = 0.924) for underweight patients and 0.71 (95% CI 0.41 to 1.23, *p* = 0.216) for overweight/obese patients. Kaplan–Meier curves similarly showed no significant differences in relapse-free survival according to baseline nutritional category (log-rank *p* = 0.75; Figure 2).

Formal assessment of potential non-linearity likewise provided no evidence that a quadratic specification improved model fit compared with the corresponding linear model for relapse rate (likelihood-ratio *p* = 0.171), time to first relapse (*p* = 0.514), or therapeutic escalation (*p* = 0.404). Results were unchanged after exclusion of the single observation with an extreme baseline BMI Z-score, with no evidence of improved fit for the quadratic specification for relapse rate (*p* = 0.478), time to first relapse (*p* = 0.961), or therapeutic escalation (*p* = 0.589). Thus, neither the categorical analyses nor the quadratic models provided evidence of a U-shaped association between baseline BMI and these medical outcomes in this cohort. As a sensitivity analysis, relapse-rate and time-to-first-relapse models were repeated after excluding the 23 patients who underwent surgical resection during follow-up. The resulting non-surgical sensitivity cohort included 160 patients, with 107 first-relapse events. The findings were broadly consistent with the primary analysis: baseline BMI Z-score was not significantly associated with relapse rate (IRR = 0.93, 95% CI 0.83 to 1.03, *p* = 0.133) or time to first relapse (HR = 0.89, 95% CI 0.79 to 1.01, *p* = 0.063). When nutritional status was analyzed categorically, underweight patients showed a higher estimated relapse rate than normal-weight patients (IRR = 1.46, 95% CI 0.92 to 2.31, *p* = 0.101) and a higher estimated hazard of first relapse (HR = 1.35, 95% CI 0.80 to 2.29, *p* = 0.263), although neither association reached statistical significance. Overweight/obesity was likewise not significantly associated with relapse rate (IRR = 0.84, 95% CI 0.52 to 1.35, *p* = 0.476) or time to first relapse (HR = 0.66, 95% CI 0.37 to 1.19, *p* = 0.168).

### 3.3. Baseline Nutritional Status and Risk of Surgical Resection

**Figure 3:**
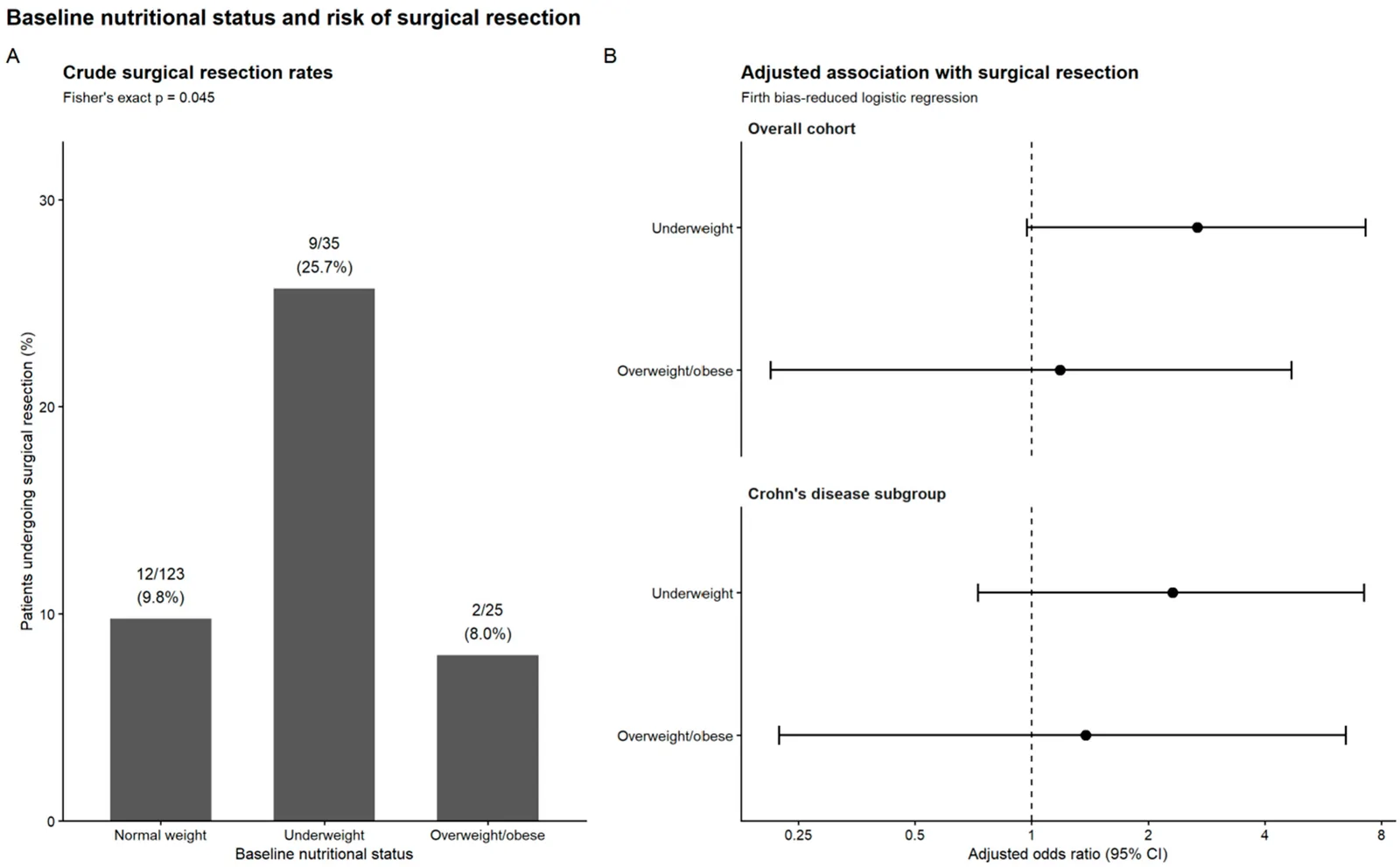
Baseline nutritional status and risk of surgical resection. (**A**) Crude proportion of patients undergoing surgical resection during follow-up according to nutritional status at diagnosis. Numbers above the bars indicate surgical events/total patients and corresponding percentages; differences between groups were assessed using Fisher’s exact test. (**B**) Adjusted odds ratios and 95% confidence intervals for surgical resection according to baseline nutritional status in the overall cohort and in the Crohn’s disease subgroup. Normal weight was the reference category. Estimates were obtained using Firth bias-reduced logistic regression. The overall-cohort model was adjusted for diagnosis, age at diagnosis, biological sex, and follow-up duration; the Crohn’s disease subgroup model was adjusted for age at diagnosis, biological sex, and follow-up duration. The dashed vertical line indicates an odds ratio of 1.

Surgical resection was performed in 23 of 183 patients (12.6%) during follow-up. Surgery rates differed across baseline nutritional categories, with resection occurring in nine of 35 underweight patients (25.7%), compared with 12 of 123 normal-weight patients (9.8%) and two of 25 overweight/obese patients (8.0%; Fisher’s exact *p* = 0.045) (see Table 4; Figure 3A).

In the exploratory Firth bias-reduced logistic regression adjusted for diagnosis, age at diagnosis, biological sex, and follow-up duration, baseline underweight showed a borderline, non-significant association with subsequent surgical resection compared with normal weight (OR = 2.68, 95% CI 0.97 to 7.27; *p* = 0.056). The confidence interval included the null value, and the global association between baseline nutritional status and surgery was not statistically significant (*p* = 0.158). Overweight/obesity was not associated with increased odds of surgery (OR = 1.19, 95% CI 0.21 to 4.69, *p* = 0.825). Diagnosis was strongly associated with surgical outcome in the adjusted model (*p* < 0.001) (see Table 4; Figure 3B).

A similar pattern was observed in the Crohn’s disease subgroup. Among 84 patients with CD, 19 underwent surgery. Resection occurred in seven of 20 underweight patients (35.0%), compared with 10 of 56 normal-weight patients (17.9%) and two of eight overweight/obese patients (25.0%), although the difference was not statistically significant (Fisher’s exact *p* = 0.281). After adjustment for age at diagnosis, biological sex, and follow-up duration, underweight patients again showed higher estimated odds of surgery than normal-weight patients (OR = 2.31, 95% CI 0.73 to 7.22, *p* = 0.152), whereas no significant association was observed for overweight/obesity (OR = 1.38, 95% CI 0.22 to 6.48, *p* = 0.702). The global association of nutritional status with surgery in the CD subgroup was not significant (*p* = 0.358).

Secondary analyses based directly on BMI Z-score thresholds yielded concordant findings. Compared with patients with BMI Z-scores between −2 and +2, those with BMI Z-score < −2 had higher estimated odds of surgery (OR = 2.64, 95% CI 0.98 to 6.93, *p* = 0.054). Only two patients had BMI Z-score > +2 and neither underwent surgery, preventing meaningful assessment of surgical risk at the upper extreme of the BMI distribution.

Baseline height Z-score was also examined as a secondary anthropometric predictor. Each 1-unit increase in height Z-score was associated with lower odds of subsequent surgical resection (OR = 0.68, 95% CI 0.47 to 1.00, *p* = 0.049). In an exploratory CD-specific model additionally accounting for disease extent, nutritional status remained non-significantly associated with surgery (global *p* = 0.289), and disease extent was not globally associated with surgical outcome (*p* = 0.148).

### 3.4. Age at Diagnosis and the VEO-IBD Phenotype

Twenty-five patients were classified as VEO-IBD on the basis of age at diagnosis < 6 years, while 158 patients were diagnosed at ≥6 years. VEO-IBD included five patients with Crohn’s disease and 20 with ulcerative colitis. Given the small size of the VEO-IBD subgroup, all analyses in this section were considered exploratory and should be interpreted cautiously. Accordingly, ulcerative colitis was more frequent among patients with VEO-IBD than among those with older-onset pediatric IBD (80.0% vs. 50.0%, *p* = 0.005). Baseline BMI Z-score did not differ significantly between groups (median −0.55 vs. −0.44, *p* = 0.332), nor did the prevalence of underweight at diagnosis (28.0% vs. 17.7%, *p* = 0.272). Median follow-up duration was longer in the VEO-IBD group (5.90 vs. 4.06 years, *p* < 0.001) (Table 5).

In disease-specific analyses, only five patients had VEO-CD, limiting inference in this subgroup. No significant differences were observed in baseline BMI Z-score, underweight prevalence, relapse rate, therapeutic escalation, or surgical resection between VEO-CD and older-onset CD. Among patients with ulcerative colitis, VEO-UC had a lower relapse rate than older-onset UC (0.38 vs. 0.59 events/person-year, *p* = 0.037), while therapeutic escalation occurred at similar frequencies (50.0% vs. 46.8%, *p* = 0.808). Surgical resection was more frequent among patients with VEO-UC (3/20, 15.0%) than among those with older-onset UC (1/79, 1.3%; *p* = 0.025) (Table 5).

**Figure 4:**
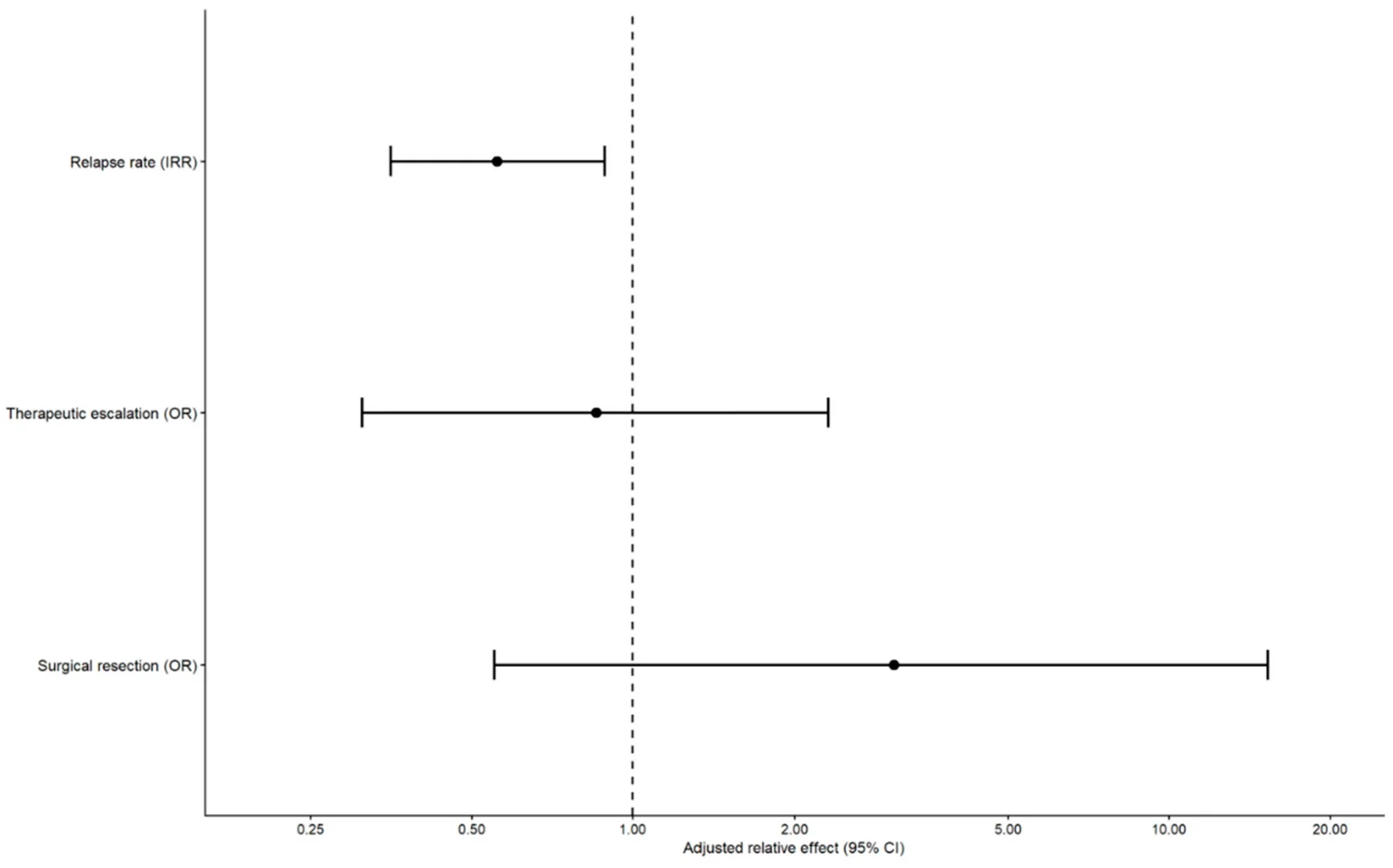
Adjusted associations between VEO-IBD status and subsequent clinical outcomes. Forest plot showing adjusted effect estimates and 95% confidence intervals for relapse rate, therapeutic escalation, and surgical resection in patients with VEO-IBD (<6 years at diagnosis) compared with older-onset pediatric IBD (≥6 years). Relapse rate was analyzed using negative binomial regression and is expressed as an incidence rate ratio (IRR); therapeutic escalation and surgical resection are expressed as odds ratios (OR), with surgical resection estimated using Firth bias-reduced logistic regression. The relapse-rate model was adjusted for diagnosis and biological sex, with follow-up duration included as an offset. Therapeutic-escalation and surgical-resection models were adjusted for diagnosis, biological sex, and follow-up duration. The dashed vertical line indicates the null value of 1.

In exploratory adjusted analyses, VEO-IBD showed a lower estimated relapse rate after accounting for diagnosis and biological sex, with follow-up duration included as an offset (IRR = 0.56, 95% CI 0.35 to 0.89; *p* = 0.013). No significant association was observed with therapeutic escalation after adjustment for diagnosis, biological sex, and follow-up duration (OR = 0.85, 95% CI 0.31 to 2.31; *p* = 0.757). For surgical resection, the adjusted point estimate was higher among patients with VEO-IBD, but the confidence interval was wide and the association was not statistically significant (OR = 3.06, 95% CI 0.55 to 15.26; *p* = 0.190) (Figure 4). Given the limited VEO-IBD sample size, these estimates should be regarded as exploratory.

### 3.5. Longitudinal Anthropometric Recovery During Follow-Up

Longitudinal BMI Z-scores were available at both diagnosis and last follow-up for 159 patients, while paired weight Z-scores were available for 157 patients. Missing T1 Z-scores occurred exclusively among patients who had reached adulthood by the last follow-up assessment. Consequently, continuous Z-score changes were analyzed for these 159 patients, whereas nutritional-category transitions were assessed for all 183 patients by applying standard adult BMI cut-offs to those who had reached adulthood at T1. Among patients with paired measurements, median BMI Z-score increased from −0.33 (IQR −1.66 to 0.42) at diagnosis to 0.27 (IQR −0.34 to 0.93) at last follow-up, corresponding to a median change of +0.54 Z-score units (IQR 0.03 to 1.51; *p* < 0.001). Weight Z-score similarly increased from −0.24 (IQR −1.10 to 0.50) to 0.16 (IQR −0.56 to 0.77), with a median change of +0.42 (IQR −0.11 to 0.91; *p* < 0.001) (Table 6).

Nutritional-category transitions were available for all 183 patients. Of the 35 patients who were underweight at diagnosis, 30 (85.7%) reached normal weight at the last follow-up, two (5.7%) remained underweight, and three (8.6%) became overweight or obese. Among the 123 patients with normal weight at diagnosis, 96 (78.0%) remained normal weight, five (4.1%) became underweight, and 22 (17.9%) became overweight or obese. Of the 25 patients who were overweight or obese at diagnosis, nine (36.0%) reached normal weight and 16 (64.0%) remained overweight or obese. Accordingly, the prevalence of underweight decreased from 35/183 (19.1%) at diagnosis to 7/183 (3.8%) at last follow-up (exact paired *p* < 0.001) (Table 6).

**Figure 5:**
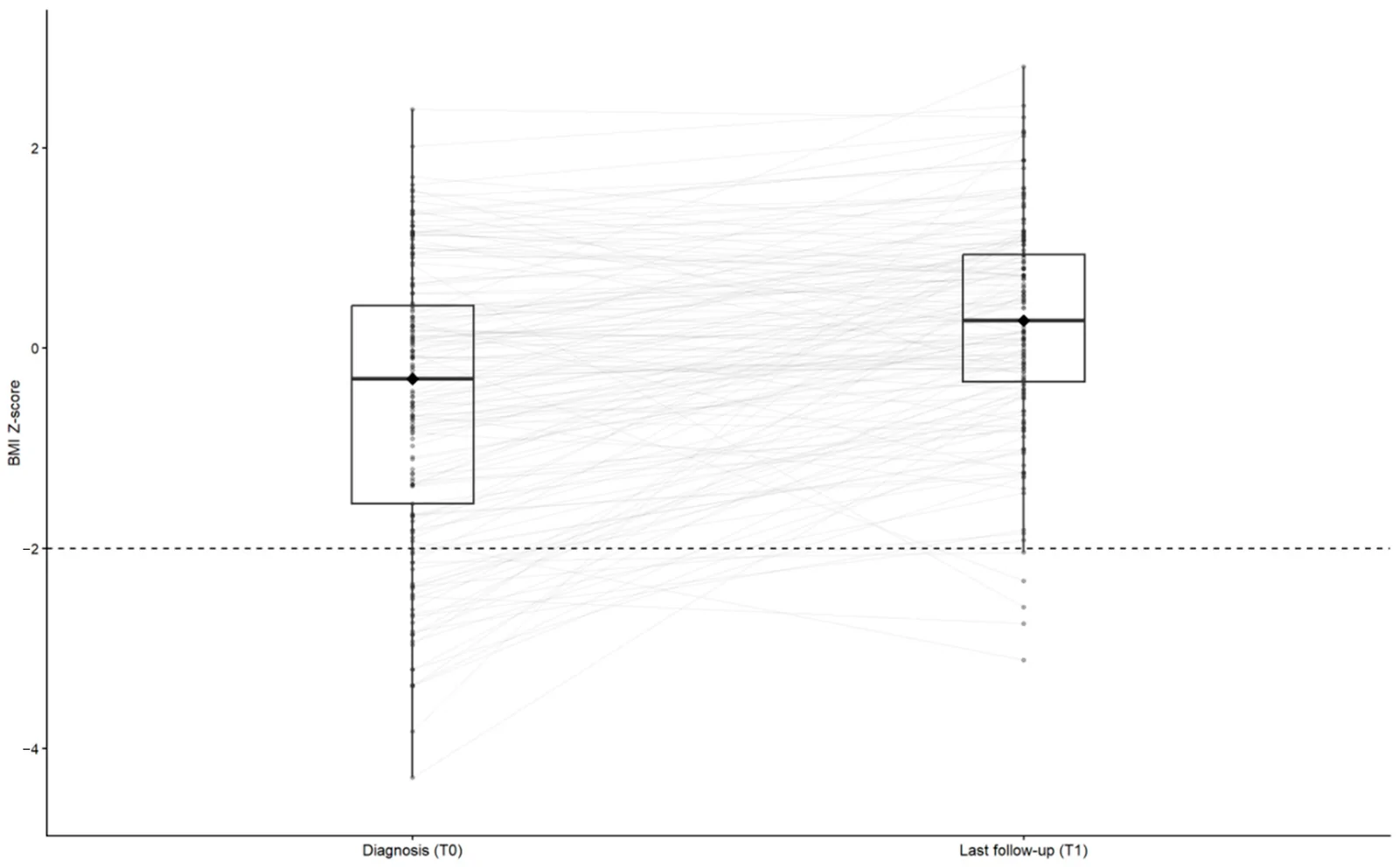
Change in BMI Z-score from diagnosis to last follow-up. Individual trajectories are shown for patients with BMI Z-score available at both time points (n = 159). Boxplots summarize the distributions at diagnosis (T0) and last follow-up (T1), with diamonds indicating the median. The dashed horizontal line represents a BMI Z-score of −2. Values outside the displayed plotting range were retained in all statistical analyses.

Individual BMI trajectories showed substantial between-patient variability, but the distribution shifted upward between diagnosis and the last follow-up, with fewer observations below the BMI Z-score threshold of −2 at T1 (Figure 5).

### 3.6. Linear Growth and Persistent Stunting

Paired height Z-scores at diagnosis and last follow-up were available for 157 patients. Median height Z-score decreased from 0.00 (IQR −0.80 to 0.69) at diagnosis to −0.25 (IQR −1.05 to 0.48) at last follow-up, corresponding to a median change of −0.14 Z-score units (IQR −0.67 to 0.19; *p* = 0.002). Despite this shift in the overall distribution, the prevalence of stunting, defined as height-for-age Z-score < −2, remained similar between diagnosis and last follow-up (6/157, 3.8% vs. 7/157, 4.5%; exact paired *p* = 1.000).

Among the 151 patients who were not stunted at diagnosis, 147 (97.4%) remained non-stunted and four (2.6%) developed new-onset stunting. Of the six patients who were stunted at diagnosis, three (50.0%) were no longer stunted at last follow-up, whereas three (50.0%) had persistent stunting. Persistent stunting therefore occurred in 3/157 patients (1.9%). Given the very small number of persistent-stunting cases, no multivariable analysis of associated factors was performed (See Table 7).

### 3.7. Clinical and Treatment-Related Factors Associated with Anthropometric Recovery

Among the 159 patients with paired BMI Z-scores, factors associated with nutritional status at last follow-up were examined using multivariable linear regression with BMI Z-score at T1 as the outcome and baseline BMI Z-score included as a covariate. Higher baseline BMI Z-score remained independently associated with higher BMI Z-score at follow-up (β = 0.31, 95% CI 0.11 to 0.51; *p* = 0.002). In contrast, total relapse burden (β = −0.04 per additional relapse, 95% CI −0.14 to 0.06; *p* = 0.443), therapeutic escalation (β = 0.12, 95% CI −0.26 to 0.50; *p* = 0.542), and surgical resection during follow-up (β = −0.04, 95% CI −0.70 to 0.63; *p* = 0.912) were not independently associated with BMI Z-score at T1. No significant associations were observed for diagnosis, sex, age at diagnosis, EIMs/comorbidities, or follow-up duration (Table 8). The multivariable model explained a modest proportion of the variability in follow-up BMI Z-score (adjusted R^2^ = 0.192).

Results were consistent in sensitivity analyses. After exclusion of the single patient with an extreme baseline BMI Z-score, baseline BMI remained associated with BMI Z-score at T1 (β = 0.40, 95% CI 0.27 to 0.53; *p* < 0.001), while none of the clinical or treatment-related factors showed an independent association with the outcome. Similarly, restricting the analysis to the 112 patients aged <18 years at T1 did not materially change the findings (baseline BMI Z-score: β = 0.34, 95% CI 0.06 to 0.61; *p* = 0.017).

When relapse burden was expressed as the number of events per person-year rather than as the total number of relapses, a higher relapse rate showed an inverse estimated association with BMI Z-score at T1 (β = −0.21, 95% CI −0.44 to 0.02; *p* = 0.070). Exploratory analyses did not support a non-linear relationship between relapse burden and growth recovery: the quadratic relapse term was not significant using HC3 robust inference (*p* = 0.411), and the categorical comparison across 0, 1, 2–5, and >5 relapses was also non-significant overall (*p* = 0.213).

## 4. Discussion

The present study provides a longitudinal assessment of nutritional status, growth, and clinical outcomes in a cohort of 183 patients with pediatric-onset IBD. Several findings emerge from the analyses. First, growth impairment at diagnosis differed according to disease phenotype, with patients with CD showing lower baseline BMI and weight Z-scores than patients with UC, while height Z-scores were comparable. Second, baseline nutritional status was not independently associated with subsequent relapse burden, time to first relapse, or therapeutic escalation. In contrast, baseline underweight was associated with a higher crude frequency of subsequent surgical resection and showed a suggestive but non-significant adjusted association with surgery (OR = 2.68, 95% CI 0.97 to 7.27; *p* = 0.056). Third, substantial growth recovery occurred during follow-up, with improvements in BMI and weight Z-scores and a marked reduction in the prevalence of underweight. Finally, BMI Z-score at diagnosis remained independently associated with BMI Z-score at last follow-up, whereas relapse burden, therapeutic escalation, surgical resection during follow-up, disease phenotype, sex, and EIMs/comorbidities were not independently associated with final BMI Z-score.

At diagnosis, underweight was present in 23.8% of patients with CD and 15.2% of those with UC, whereas overweight or obesity was observed in 9.5% and 17.2%, respectively. The lower BMI and weight Z-scores observed in CD are consistent with previous pediatric studies reporting a greater burden of growth impairment in CD than in UC [3,4,5]. Several mechanisms may contribute to this difference, including reduced oral intake, systemic inflammation, increased energy expenditure, and, particularly in CD, small-bowel involvement and malabsorption [2,17]. However, anatomical disease extent was not independently associated with baseline BMI Z-score in either CD or UC in our cohort. This suggests that the nutritional phenotype at diagnosis cannot be explained solely by the anatomical distribution of intestinal involvement. Age at diagnosis was also positively associated with BMI Z-score, particularly within UC, indicating that younger children may present with a less favorable nutritional profile, although the magnitude of this association was modest.

An important finding of the present study is the absence of a clear independent relationship between baseline BMI and subsequent medical disease instability. Neither BMI Z-score as a continuous measure nor baseline nutritional categories were significantly associated with relapse rate, time to first relapse, or therapeutic escalation. These findings were maintained after exclusion of patients who underwent surgical resection. They therefore do not support the hypothesis that the absence of an association in the overall cohort resulted from preferential removal of more severe patients from subsequent relapse follow-up through surgery. Previous studies have reported associations between both low and high BMI and a more severe pediatric IBD course, leading to the proposal of a U-shaped relationship between BMI and adverse outcomes [6,7]. However, other pediatric cohorts focusing on overweight and obesity have reported more heterogeneous associations with dis-ease activity and complications [8]. In our cohort, analyses based on baseline nutritional categories did not show significant associations of either underweight or overweight/obesity with relapse rate, time to first relapse, or therapeutic escalation. Formal assessment of non-linearity yielded consistent findings, as addition of a quadratic baseline BMI Z-score term did not significantly improve model fit for any of these three outcomes. These results were unchanged after exclusion of the single extreme baseline BMI Z-score observation. Thus, within the limitations of the present sample, our findings do not provide evidence for the U-shaped association between baseline BMI and subsequent medical disease instability proposed in previous studies. Separately, exploratory analyses examining relapse burden in relation to anthropometric recovery did not support either a quadratic or categorical non-linear pattern. Differences in study population, outcome definitions, duration of follow-up, therapeutic strategies, and the limited number of patients at the extreme upper end of the BMI distribution may partly account for these discrepancies.

Conversely, disease phenotype was more strongly associated with medical disease course than baseline nutritional status. Patients with UC had both a higher adjusted relapse rate and a higher hazard of first relapse than patients with CD. Older age at diagnosis was also associated with a modest increase in relapse rate. These findings suggest that anthropometric status at presentation and subsequent inflammatory activity represent related but not interchangeable dimensions of pediatric IBD severity. Baseline BMI may reflect the cumulative nutritional and inflammatory burden preceding diagnosis, whereas subsequent relapse behavior may be determined by additional disease-specific biological and therapeutic factors.

The relationship between baseline nutritional status and surgery was different from that observed for medical outcomes. Surgical resection occurred in 25.7% of underweight patients, compared with 9.8% of normal-weight patients and 8.0% of overweight/obese patients. After adjustment, baseline underweight showed a borderline, non-significant association with subsequent surgery compared with normal weight (OR = 2.68, 95% CI 0.97 to 7.27; *p* = 0.056). This finding should not be interpreted as evidence that underweight is an independent predictor of surgery. Rather, it should be regarded as an exploratory, suggestive signal that requires validation in larger prospective cohorts. A similar direction of association was observed in the CD subgroup. These results should therefore not be interpreted as demonstrating that underweight independently predicts surgery, but they suggest that growth impairment at diagnosis may identify a subgroup with a greater burden of disease eventually requiring surgical management. Previous work has identified growth impairment and markers of complicated disease as relevant prognostic features in pediatric CD [10], while diagnostic delay has been associated with both complicated disease behavior and impaired growth [11]. The additional association observed between higher baseline height Z-score and lower odds of surgery is compatible with the possibility that cumulative disease burden before diagnosis may be reflected in multiple dimensions of growth and nutritional status. Nevertheless, this secondary finding was borderline and requires confirmation in larger cohorts. Evidence, predominantly from adult IBD populations, suggests that body-composition measures such as lean mass or sarcopenia, which are not captured by BMI alone, may provide additional prognostic information [18].

The longitudinal analyses demonstrated substantial recovery of weight-related nutritional indices. Among patients with paired data, median BMI Z-score increased by 0.54 units and weight Z-score by 0.42 units. Moreover, 30 of the 35 patients who were underweight at diagnosis reached normal weight by the last follow-up, and the overall prevalence of underweight decreased from 19.1% to 3.8%. These findings illustrate the considerable capacity for growth recovery during pediatric IBD follow-up. Importantly, however, recovery was not uniform: some initially normal-weight patients subsequently became overweight or obese, and almost two-thirds of patients who were overweight or obese at diagnosis remained in the upper nutritional categories. Nutritional surveillance should therefore address both insufficient and excessive weight gain rather than focusing exclusively on undernutrition.

The pattern for linear growth was more complex. Although median height Z-score showed a small but statistically significant decrease during follow-up, the prevalence of stunting remained essentially unchanged, and persistent stunting occurred in only three patients. Thus, the observed shift in the overall height Z-score distribution did not translate into a substantial increase in clinically defined stunting. Growth impairment in pediatric IBD is multifactorial and may be influenced by inflammatory activity, nutritional status, endocrine pathways, corticosteroid exposure, disease duration, and pubertal development [2,12,13,14]. Because pubertal stage and growth velocity were not systematically available in the present cohort, the observed longitudinal change in height Z-score cannot be attributed to pubertal timing or to any specific biological mechanism. The small number of patients with persistent stunting also precluded a meaningful analysis of its predictors.

Age at disease onset provided an additional exploratory perspective on clinical heterogeneity. VEO-IBD accounted for only 25 patients, including five with CD, substantially limiting the precision and reliability of subgroup inference. In exploratory adjusted analyses, VEO-IBD showed a lower estimated relapse rate, whereas no significant associations were identified with therapeutic escalation or surgical resection. These findings should be interpreted cautiously and require confirmation in larger cohorts. Disease-specific analyses were even more limited. Within UC, VEO-UC patients had a lower relapse rate but a higher crude frequency of surgery than older-onset UC; however, only four surgical events occurred in the entire UC comparison, three of them among VEO-UC patients. The overall adjusted estimate for surgery in VEO-IBD was imprecise and non-significant. These findings should therefore not be interpreted as evidence of a distinct prognostic phenotype. The marked biological heterogeneity of VEO-IBD, including the recognized contribution of monogenic and immune dysregulation disorders in selected patients, remains clinically relevant and supports dedicated diagnostic assessment in appropriate cases [15]. An additional finding concerns the relationship between treatment course and anthropometric recovery. Therapeutic escalation was not independently associated with BMI Z-score at last follow-up after accounting for baseline BMI Z-score, relapse burden, diagnosis, age, sex, EIMs/comorbidities, and follow-up duration. Similarly, total relapse burden was not independently associated with final BMI Z-score in the primary model. When relapse burden was expressed as events per person-year, the estimated association was inverse but did not reach statistical significance. Because therapeutic escalation in our database was heterogeneous and could include azathioprine as well as biologic therapy, and because treatment allocation was not randomized, the present data cannot be used to estimate the causal effect of individual therapeutic strategies on growth recovery. Current ECCO-ESPGHAN guidance for pediatric Crohn’s disease recognizes severe growth retardation as a high-risk feature and incorporates nutritional therapy and growth considerations into disease management [19]. Comparative effects of individual therapeutic strategies on anthropometric recovery, however, would require specifically designed prospective analyses.

The association between baseline and follow-up BMI Z-score also deserves consideration. Even after adjustment for clinical course and treatment-related factors, higher BMI Z-score at diagnosis was associated with higher BMI Z-score at follow-up. However, the multivariable model explained only a modest proportion of the variability in follow-up BMI Z-score (adjusted R^2^ = 0.192). Baseline BMI should therefore not be interpreted as determining subsequent growth recovery. At the population level, substantial catch-up occurred, particularly among initially underweight patients, while considerable between-patient variability remained. This residual variability may reflect factors not captured in our model, including pre-disease growth trajectory, dietary intake, body composition, pubertal development, and other unmeasured biological factors.

Anthropometric recovery should also not be considered synonymous with complete anthropometric recovery. BMI and weight Z-scores do not capture body composition or micronutrient status. Iron, vitamin D, zinc, and other micronutrient deficiencies are common in pediatric IBD and may persist during follow-up despite improvement in anthropometric status [17,20,21]. Consequently, the marked reduction in underweight observed in our cohort should not be interpreted as evidence that all nutritional abnormalities had resolved. Continued assessment of micronutrient status and body composition, where clinically indicated, remains complementary to routine anthropometric monitoring.

Several limitations should be acknowledged. First, the retrospective, single-center design limits generalizability and precludes causal inference. Furthermore, due to the retrospective nature of the study, baseline clinical activity indices and complete inflammatory markers were not uniformly available for all patients at diagnosis. As a result, disease severity and inflammatory burden could not be included as covariates in our baseline multivariable models. This represents a significant limitation, as it is difficult to fully untangle whether baseline growth impairment independently influences subsequent disease course or if it merely acts as a surrogate marker for a more aggressive baseline inflammatory phenotype. Second, some subgroup analyses involved very small numbers, particularly the VEO-IBD analyses and, within them, the VEO-CD subgroup, as well as the upper extreme of the BMI distribution and analyses of persistent stunting and surgical outcomes within UC; these results should therefore be regarded as exploratory. Third, BMI is an indirect measure of nutritional status and does not distinguish fat mass from lean mass or identify sarcopenia. Micronutrient and biochemical nutritional markers were not systematically available. Fourth, BMI, weight, and height Z-scores at T1 were unavailable for some patients who had reached adulthood, although the sensitivity analysis restricted to patients aged <18 years at T1 yielded results consistent with the primary growth-recovery model. Fifth, pubertal stage, longitudinal growth velocity, and cumulative corticosteroid exposure were not systematically recorded. These factors can act as significant residual confounders, limiting the mechanistic interpretation of changes in height and BMI Z-scores. Moreover, the composite variable used for EIMs and comorbidities grouped a heterogeneous range of conditions. While this approach was necessary due to the limited sample size of individual pathologies, we acknowledge that different comorbidities might exert distinct biological influences on growth and nutritional trajectories, potentially introducing a degree of residual confounding. Sixth, the analytical dataset recorded whether surgical resection occurred but did not include the exact date of surgery. Therefore, relapse follow-up could not be censored at the time of resection, and the influence of surgery on relapse ascertainment was instead examined by excluding surgically treated patients in a sensitivity analysis. Finally, therapeutic escalation was observational, heterogeneous, and influenced by clinical indication, preventing causal comparisons between specific treatment strategies.

## 5. Conclusions

In this longitudinal cohort of patients with pediatric-onset IBD, nutritional status at diagnosis differed according to disease phenotype, with greater weight- and BMI-related impairment in CD than in UC. Baseline nutritional status was not independently associated with subsequent relapse burden, time to first relapse, or therapeutic escalation, suggesting that anthropometric impairment at diagnosis has limited value as a predictor of subsequent medical disease instability. In contrast, baseline underweight showed a higher crude frequency of surgical resection and a suggestive but non-significant adjusted association with surgery, indicating a potential signal that warrants confirmation in larger cohorts.

Growth recovery during follow-up was substantial, with marked improvements in BMI and weight Z-scores and a reduction in underweight prevalence from 19.1% to 3.8%, while persistent stunting was uncommon. At the individual level, baseline BMI Z-score was associated with BMI Z-score at follow-up, whereas relapse burden and therapeutic escalation were not independently associated with nutritional outcome. However, the multivariable model explained only a modest proportion of between-patient variability, indicating that long-term auxological recovery is likely influenced by additional factors not captured in the present analysis. These findings support longitudinal nutritional and growth monitoring as an integral component of pediatric IBD care, while highlighting the need for larger prospective studies to clarify the prognostic significance of baseline undernutrition and the determinants of long-term nutritional recovery.

## Figures and Tables

**Table 1:** Demographic, anthropometric, and clinical characteristics of the study cohort. Continuous variables are reported as median (IQR) and categorical variables as n (%). Comparisons between Crohn’s disease (CD) and ulcerative colitis (UC) were performed using the Wilcoxon rank-sum test for continuous variables and Fisher’s exact test for categorical variables. BMI, body mass index; CD, Crohn’s disease; UC, ulcerative colitis.

Characteristic	Total (N = 183)	CD (N = 84)	UC (N = 99)	*p*-Value
**Sex**				0.234
Female	81 (44.3%)	33 (39.3%)	48 (48.5%)	
Male	102 (55.7%)	51 (60.7%)	51 (51.5%)	
**Age at diagnosis, years**	12.00 (9.00, 14.00)	13.00 (10.00, 14.00)	11.00 (6.00, 14.00)	0.048
**Weight at diagnosis, kg**	40.00 (25.50, 51.40)	37.50 (28.68, 49.73)	42.80 (23.00, 53.75)	0.923
**Height at diagnosis, cm**	151.90 (133.00, 164.25)	151.95 (136.00, 165.00)	151.00 (121.50, 163.00)	0.230
**BMI at diagnosis, kg/m^2^**	16.90 (15.20, 19.75)	16.05 (14.80, 18.55)	17.50 (15.60, 20.65)	0.019
**Weight Z-score at T0**	−0.34 (−1.15, 0.41)	−0.68 (−1.39, 0.06)	−0.04 (−0.75, 0.58)	0.001
**Height Z-score at T0**	−0.10 (−0.83, 0.69)	−0.16 (−1.01, 0.59)	0.08 (−0.70, 0.87)	0.144
**BMI Z-score at T0**	−0.45 (−1.66, 0.38)	−0.82 (−1.94, 0.05)	−0.06 (−0.96, 0.67)	<0.001
**Nutritional status at T0**				0.208
Normal weight	123 (67.2%)	56 (66.7%)	67 (67.7%)	
Underweight	35 (19.1%)	20 (23.8%)	15 (15.2%)	
Overweight	23 (12.6%)	7 (8.3%)	16 (16.2%)	
Obese	2 (1.1%)	1 (1.2%)	1 (1.0%)	
**Surgical resection during follow-up**				<0.001
No	160 (87.4%)	65 (77.4%)	95 (96.0%)	
Yes	23 (12.6%)	19 (22.6%)	4 (4.0%)	

**Table 2:** Multivariable linear regression analysis of factors associated with BMI Z-score at diagnosis. β coefficients represent adjusted differences in baseline BMI Z-score. CI, confidence interval; CD, Crohn’s disease; UC, ulcerative colitis; EIMs, extraintestinal manifestations.

Characteristic	β	95% CI	*p*-Value
Panel A. Overall cohort (N = 183)			
UC vs. CD	0.66	0.20 to 1.12	**0.005**
Male vs. female	−0.31	−0.77 to 0.15	0.181
Age at diagnosis, per year	0.06	0.01 to 0.12	**0.028**
EIMs/comorbidities, yes vs. no	−0.01	−0.47 to 0.44	0.951
Panel B. Crohn’s disease (N = 84)			
Disease extent, global	—	—	0.603
Ileal vs. colonic/pancolitis	0.01	−0.88 to 0.91	0.975
Upper GI vs. colonic/pancolitis	0.35	−0.62 to 1.32	0.475
Male vs. female	−0.32	−0.95 to 0.30	0.306
Age at diagnosis, per year	0.04	−0.06 to 0.14	0.470
EIMs/comorbidities, yes vs. no	−0.39	−1.02 to 0.23	0.215
Panel C. Ulcerative colitis (N = 99)			
Extensive/colonic vs. distal	−0.49	−1.28 to 0.31	0.230
Male vs. female	−0.36	−1.03 to 0.31	0.286
Age at diagnosis, per year	0.09	0.01 to 0.17	0.021
EIMs/comorbidities, yes vs. no	0.37	−0.31 to 1.05	0.283

**Table 3:** Prognostic value of baseline BMI Z-score for subsequent medical outcomes. Estimates refer to a 1-unit increase in baseline BMI Z-score. Models were adjusted for diagnosis, biological sex, age at diagnosis, and EIMs/comorbidities. The relapse-rate model accounted for follow-up duration using an offset, whereas follow-up duration was additionally included as a covariate in the therapeutic-escalation model. The Cox model included 183 patients and 123 first-relapse events. BMI, body mass index; IRR, incidence rate ratio; HR, hazard ratio; OR, odds ratio; CI, confidence interval.

Outcome	Model	N	Effect Measure	Estimate	95% CI	*p*-Value
Relapse rate	Negative binomial regression	183	IRR	0.94	0.84 to 1.05	0.221
Time to first relapse	Cox proportional hazards	183	HR	0.93	0.83 to 1.05	0.234
Therapeutic escalation	Logistic regression	183	OR	1.11	0.90 to 1.38	0.351

**Table 4:** Association between baseline nutritional status and risk of surgical resection. Adjusted odds ratios were estimated using Firth bias-reduced logistic regression. The overall-cohort model was adjusted for diagnosis, age at diagnosis, biological sex, and follow-up duration. The Crohn’s disease subgroup model was adjusted for age at diagnosis, biological sex, and follow-up duration. Normal weight was the reference category. OR, odds ratio; CI, confidence interval.

Analysis	Baseline Nutritional Status	Surgery, n/N (%)	Adjusted OR	95% CI	*p*-Value
Overall cohort (global p = 0.158)					
	Normal weight	12/123 (9.8%)	1.00	Reference	—
	Underweight	9/35 (25.7%)	2.68	0.97–7.27	0.056
	Overweight/obese	2/25 (8.0%)	1.19	0.21–4.69	0.825
Crohn’s disease subgroup (global p = 0.358)					
	Normal weight	10/56 (17.9%)	1.00	Reference	—
	Underweight	7/20 (35.0%)	2.31	0.73–7.22	0.152
	Overweight/obese	2/8 (25.0%)	1.38	0.22–6.48	0.702

**Table 5:** Clinical characteristics and outcomes according to age at diagnosis. VEO-IBD was defined as age at diagnosis < 6 years; patients diagnosed at age ≥ 6 years were classified as older-onset pediatric IBD. Continuous variables are reported as median (IQR) and were compared using the Wilcoxon rank-sum test. Categorical variables were compared using Fisher’s exact test. Relapse rates were calculated as the total number of relapses divided by accumulated person-years of follow-up, with *p*-values obtained from negative binomial regression using log follow-up duration as an offset. All comparisons involving VEO-IBD should be interpreted as exploratory because of the limited subgroup size; disease-specific comparisons require additional caution, particularly for Crohn’s disease because only five patients had VEO-CD.

Characteristic	VEO-IBD (<6 Years)	Older-Onset Pediatric IBD (≥6 years)	*p*-Value
A. Overall cohort			
Patients, n	25	158	—
Ulcerative colitis, n/N (%)	20/25 (80.0%)	79/158 (50.0%)	0.005
Baseline BMI Z-score, median (IQR)	−0.55 (−2.46 to 0.22)	−0.44 (−1.55 to 0.44)	0.332
Underweight at diagnosis, n/N (%)	7/25 (28.0%)	28/158 (17.7%)	0.272
Follow-up duration, years, median (IQR)	5.90 (4.39 to 10.47)	4.06 (2.44 to 5.45)	<0.001
Relapse rate, events/person-year	0.30	0.42	0.114
Therapeutic escalation, n/N (%)	14/25 (56.0%)	68/158 (43.0%)	0.280
Surgical resection, n/N (%)	3/25 (12.0%)	20/158 (12.7%)	1.000
B. Crohn’s disease			
Patients, n	5	79	—
Baseline BMI Z-score, median (IQR)	−0.57 (−2.86 to −0.11)	−0.84 (−1.91 to 0.06)	0.664
Underweight at diagnosis, n/N (%)	2/5 (40.0%)	18/79 (22.8%)	0.588
Follow-up duration, years, median (IQR)	13.02 (12.67 to 13.74)	4.42 (2.82 to 5.88)	<0.001
Relapse rate, events/person-year	0.14	0.28	0.162
Therapeutic escalation, n/N (%)	4/5 (80.0%)	31/79 (39.2%)	0.155
Surgical resection, n/N (%)	0/5 (0.0%)	19/79 (24.1%)	0.583
C. Ulcerative colitis			
Patients, n	20	79	—
Baseline BMI Z-score, median (IQR)	−0.41 (−2.00 to 0.22)	0.04 (−0.83 to 0.84)	0.097
Underweight at diagnosis, n/N (%)	5/20 (25.0%)	10/79 (12.7%)	0.177
Follow-up duration, years, median (IQR)	5.14 (4.21 to 7.83)	3.84 (2.37 to 5.23)	0.005
Relapse rate, events/person-year	0.38	0.59	0.037
Therapeutic escalation, n/N (%)	10/20 (50.0%)	37/79 (46.8%)	0.808
Surgical resection, n/N (%)	3/20 (15.0%)	1/79 (1.3%)	0.025

**Table 6:** Longitudinal changes in nutritional parameters. Continuous analyses included patients with measurements available at both diagnosis (T0) and last follow-up (T1) and are reported as median (IQR). Change was calculated as T1 minus T0, and *p*-values were obtained using the paired Wilcoxon signed-rank test. In Panel B, cells show n (row %), representing nutritional status at T1 within each baseline nutritional category. Overweight and obesity were combined into a single category. In Panel B, categorical transitions are reported for the entire cohort (N = 183); for the 24 patients who reached adulthood at T1, nutritional status was defined using standard adult BMI cut-offs (<18.5, 18.5–24.9, and ≥25.0 kg/m^2^).

Panel/Parameter	Paired N	T0, Median (IQR)	T1, Median (IQR)	Change T1 − T0, Median (IQR)	*p*-Value
Panel A. Changes in continuous nutritional parameters					
BMI Z-score	159	−0.33 (−1.66 to 0.42)	0.27 (−0.34 to 0.93)	0.54 (0.03 to 1.51)	<0.001
Weight Z-score	157	−0.24 (−1.10 to 0.50)	0.16 (−0.56 to 0.77)	0.42 (−0.11 to 0.91)	<0.001
Panel B. Nutritional-category transitions from diagnosis to last follow-up					
Nutritional status at T0		Underweight at T1	Normal weight at T1	Overweight/obese at T1	
Underweight		2 (5.7%)	30 (85.7%)	3 (8.6%)	
Normal weight		5 (4.1%)	96 (78.0%)	22 (17.9%)	
Overweight/obese		0 (0.0%)	9 (36.0%)	16 (64.0%)	

**Table 7:** Longitudinal changes in linear growth and stunting status. Continuous variables are reported as median (IQR). Analyses were restricted to patients with height Z-score available at both diagnosis (T0) and last follow-up (T1). Change was calculated as T1 minus T0 and was assessed using the paired Wilcoxon signed-rank test. Stunting was defined as height-for-age Z-score < −2. The change in stunting prevalence was assessed using an exact paired test based on discordant pairs. In Panel B, cells show n (row %). Persistent stunting was defined as stunting at both T0 and T1.

Panel/Outcome	Paired N	T0	T1	Change T1 − T0	*p*-Value
Panel A. Longitudinal linear growth					
Height Z-score, median (IQR)	157	0.00 (−0.80 to 0.69)	−0.25 (−1.05 to 0.48)	−0.14 (−0.67 to 0.19)	0.002
Stunting, n/N (%)	157	6/157 (3.8%)	7/157 (4.5%)	—	1.000
Panel B. Height-status transitions from diagnosis to last follow-up					
Height status at T0		Non-stunted at T1	Stunted at T1		
Non-stunted		147 (97.4%)	4 (2.6%)		
Stunted		3 (50.0%)	3 (50.0%)		

**Table 8:** Multivariable linear regression with BMI Z-score at last follow-up (T1) as the dependent variable. Baseline BMI Z-score, total relapse burden, therapeutic escalation, surgical resection during follow-up, diagnosis, biological sex, age at diagnosis, EIMs/comorbidities, and follow-up duration were entered simultaneously. β coefficients are ordinary least-squares point estimates; 95% confidence intervals and *p*-values are based on HC3 heteroskedasticity-robust standard errors. Therapeutic escalation was defined as any recorded escalation code 1–3 versus 0; because the source treatment variable may also include azathioprine, it is not labeled as biologic therapy. N = 159; adjusted R^2^ = 0.192.

Factor	β	95% CI	*p*-Value
Baseline BMI Z-score, per 1-unit increase	0.31	0.11 to 0.51	0.002
Total relapse burden, per additional relapse	−0.04	−0.14 to 0.06	0.443
Therapeutic escalation, yes vs. no	0.12	−0.26 to 0.50	0.542
Surgical resection during follow-up, yes vs. no	−0.04	−0.70 to 0.63	0.912
Ulcerative colitis vs. Crohn’s disease	−0.10	−0.48 to 0.29	0.623
Male vs. female	−0.11	−0.43 to 0.22	0.517
Age at diagnosis, per year	−0.05	−0.10 to 0.01	0.104
EIMs/comorbidities, yes vs. no	−0.02	−0.33 to 0.30	0.914
Follow-up duration, per year	−0.04	−0.13 to 0.05	0.356

## Data Availability

The data presented in this study are available on reasonable request from the corresponding author. They are not publicly available due to privacy.
